# Realizing IoT service’s policy privacy over publish/subscribe-based middleware

**DOI:** 10.1186/s40064-016-3250-x

**Published:** 2016-09-20

**Authors:** Li Duan, Yang Zhang, Shiping Chen, Shiyao Wang, Bo Cheng, Junliang Chen

**Affiliations:** 1 State Key Laboratory of Networking and Switching Technology, Beijing University of Posts and Telecommunications, Beijing, 100876 China; 2 Data61, CSIRO, Marsfield, NSW 2122 Australia

**Keywords:** IoT service, Publish/subscribe paradigm, Middleware, Policy privacy framework, Access control

## Abstract

The publish/subscribe paradigm makes IoT service collaborations more scalable and flexible, due to the space, time and control decoupling of event producers and consumers. Thus, the paradigm can be used to establish large-scale IoT service communication infrastructures such as Supervisory Control and Data Acquisition systems. However, preserving IoT service’s policy privacy is difficult in this paradigm, because a classical publisher has little control of its own event after being published; and a subscriber has to accept all the events from the subscribed event type with no choice. Few existing publish/subscribe middleware have built-in mechanisms to address the above issues. In this paper, we present a novel access control framework, which is capable of preserving IoT service’s policy privacy. In particular, we adopt the publish/subscribe paradigm as the IoT service communication infrastructure to facilitate the protection of IoT services policy privacy. The key idea in our policy-privacy solution is using a two-layer cooperating method to match bi-directional privacy control requirements: (a) data layer for protecting IoT events; and (b) application layer for preserving the privacy of service policy. Furthermore, the anonymous-set-based principle is adopted to realize the functionalities of the framework, including policy embedding and policy encoding as well as policy matching. Our security analysis shows that the policy privacy framework is Chosen-Plaintext Attack secure. We extend the open source Apache ActiveMQ broker by building into a policy-based authorization mechanism to enforce the privacy policy. The performance evaluation results indicate that our approach is scalable with reasonable overheads.

## Background

A distributed event-based publish/subscribe system is an asynchronous message communication paradigm, in which the space, the time and the control are decoupled between event producers (publishers) and event consumers (subscribers) (Eugster et al. 2003; Robinson and Clark 2010). For this reason, the paradigm can be used to establish an appropriate communication infrastructure for Internet of Things (IoT) services to collaborate with each other over the internet (Li et al. 2010; Al-Fuqaha et al. 2015). For example, Supervisory Control and Data Acquisition (SCADA) systems are designed to act as the core of the power grid, and can interact with other services without side effects. Considerable efforts have been made to standardise the publish/subscribe service functionalities and APIs, such as Data Distribution Service (DDS) issued by OMG^1^ and Java Message Service (JMS).^2^

The GridStat (Washington State University) project (Bakken et al. 2011) adopted the publish/subscribe paradigm to build their communication infrastructure, where the data consumers can express their interests through subscription without knowing who produces the data. On the other hand, the data producer publishes their data without knowing who subscribes to the data, which means that the data interactions within the publish/subscribe paradigm are anonymous. Such communication infrastructure no longer cares for “where” the information is located, but “what” information is needed. A customer is able to describe his/her requirements by “event type”, and the infrastructure will deliver data with the “type” to him/her, even if the data producer does not intend to send the data to the customer (Eugster 2007). Multiple customers are allowed to subscribe to the “same type” data, which implies that multicast is an intrinsic method of communication in the infrastructure (Hosseini et al. 2007). The publish/subscribe paradigm also removes the conversation restriction (Paci et al. 2011): no communication source and destination are needed, and the event types are able to be hierarchically structured. In addition, automatic caching is enabled by event type. Since each data packet is meaningful and independent of its provenance or where it may be forwarded to, it can be cached in a real-time database to satisfy future requirements. Although the GridStat^3^ communication architecture provided basic publish/subscribe messaging capability for the smart grid, it did not thoroughly address the security issues in its infrastructure. Moreover, the GridStat project did not discuss its impact on services and events.

However, with IoT systems becoming open and wide-area, various IoT services in different domains collaborate with each other for their mutual interests. In contrast to service orchestration in SOA middleware (Loyall et al. 2011), IoT service interactions rely on publish/subscribe-based middleware, which is used to carry out event data routing and matching. In this scenario, some sensitive information may be eavesdropped on pervasive service interactions. Thus, it is desirable to protect sensitive information between subscribers (or users)and publishers (or enterprises) from unauthorized access (Wang et al. 2014; Srivatsa and Liu 2005).

In particular, the privacy requirements related to service interactions can be divided into two major aspects (Wang et al. 2002; Fiege et al. 2004; Esposito and Ciampi 2015): (1) data policy privacy–data publishers do not like other unauthorized subscribers to access their published data, that is to say, only authorized subscribers can read the events; (2) service policy privacy–a subscriber may intend to maintain privacy. Our work here is concerned with how to preserve the policy privacy so as to manage events and services security within publish/subscribe-based IoT service interactions.

To preserve IoT service’s policy privacy is difficult in the publish/subscribe paradigm, due to the anonymity, multicast and indirection properties of service interactions. Attribute-based access control is one of the most popular access control models (Hu et al. 2015), which can be used to preserve the anonymity property. Embedding the policies and attributes into data and services makes services interact using a data-centric methodology, which can be used to preserve the multicast property. The main challenge is how to comprehensively preserve the policy privacy of data and services using policy matching. Bi-directional privacy policy matching means that any published data can only be sent to authorized users who are interested in it. In other words, a broker needs to check whether the published data’s attributes satisfy the subscription policy provided by the subscribers, i.e., whether subscribers are interested in the data. In the meantime, the broker needs to check whether the attributes of the subscriber satisfy the access policy related to the published data, i.e., whether the data can be received by the subscriber. The direct matching will result in privacy information leakage, an attribute blinding approach can be used to address this problem. In previous work, there have been some policy privacy approaches that allow the brokers to check whether the attributes of the consumers satisfy the access policy. However, to the best of our knowledge, few existing approaches can support a comprehensive protection of data policy privacy and services policy privacy.

In this paper, we adopt the publish/subscribe paradigm as an IoT service communication infrastructure, whose underlying network capabilities can be integrated to facilitate policy-aware messaging between IoT services. To preserve policy privacy, we present a novel policy privacy model, namely a two-layer access control framework. The key point in our policy-privacy solution is using a two-layer cooperating method to meet the bi-directional privacy control requirements, which can support two-layer policy privacy: (1) the bottom one is the data layer for protecting data or events; (2) the upper one is the application layer for protecting services. The framework addresses the issues of preserving IoT service’s policy privacy using a data-centric methodology. Furthermore, the policy bedding function, encoding and blinding functions are realized by applying the anonymous-set-based principle to preserve policy privacy. Such encoding and blinding attributes are Chosen-Plaintext Attack (CPA) secure, in which the same attribute under two different encodings and blinding will generate two different encoded and blinded attributes. Later, we choose one of the publish/subscribe service standards JMS to implement our access control framework. Apache ActiveMQ is used as the JMS broker and extended to perform policy evaluation. The main contributions of this paper are as follows:

1. A publish/subscribe-based IoT service communication infrastructure is modelled.
2. A two-layer access control framework for IoT services is proposed to allow publishers and subscribers to control the messaging data by matching between protection requirements and entities’ capabilities.
3. Two key components are designed to act as the corner stones of the framework: (1) the policy embedding component where the policy and attributes can be dynamically generated and embedded; and (2) the blind encoding component for polices and attributes of IoT events, which realizes policy privacy. The anonymous-set-based principle is adopted to assist realizing their functions.

The remainder of the paper is organized as follows. In Sect. “Related work”, we review the existing work related to our work. Section “Preliminarie” introduces the basic Publish/Subscribe-based IoT’s service (i.e., SCADA) communication infrastructure and the generic concepts used in our approach. Section “Access control framework for SCADA systems” presents our access control framework for SCADA systems. Section “Policy embedding scheme” provides an embedding scheme for realizing the matching function in our access control framework. Section “Policy encoding and matching” goes into detail about policy encoding and matching to enforce access control policy. Section “Policy privacy” presents the security analysis and proof so as to ensure the correctness of our approaches. Section “Storage cost and performance evaluation” presents the storage cost and performance evaluation on Latency. Section “Conclusions and future research” provides conclusions and outlines future research.

## Related work

There has been considerable work on policy privacy of secure service interactions over publish/subscribe-based systems. In this section, we will discuss related work in the following aspects:

### Privacy preserving technique

The cryptographic encryption solution is a common privacy-preserving technique used in the distributed system (Goyal et al. 2006; Waters 2011; Masaud-Wahaishi and Gaouda 2011; Nishide and Yoneyama 2009; Cheung and Newport 2007; Wang et al. 2014). Goyal et al. (2006) provided a Key-Policy ABE scheme, which allowed the policies (attached to keys) to be expressed by any monotonic formula over encrypted attributes (ciphertext). Waters (2011) proposed the Ciphertext-Policy Attribute Encryption (CP-ABE) scheme, where any encryptor was allowed to specify access control in terms of any access formula over the attributes in the system. However, in these approaches, the CP-ABE scheme embeds authorization policies into ciphertexts. Such schemes in publish/subscribe systems require that a participant have many keys, where each publisher gives the participant a key. It does not allow for using notification brokers to reduce the key management burden of the participant, and does not preserve the decoupling feature between service providers and consumers, which cannot assure the expression power of broker-integrable policies. Other studies have Yu et al. (2008), Li et al. (2012), Doshi and Jinwala (2011), Müller and Katzenbeisser (2011) also proposed a policy-privacy attribute-based encryption scheme, where authorization policies were hidden within the ciphertexts as well as reducing the size of the ciphertexts. These works focused on hiding policies into ciphertexts similar to policy encryption, but did not focus on the policy anonymity approach based on anonymous sets and support to manage policies flexibly. In SCADA scenarios, the authorization policies for long existing event types may be possibly modified. Updating authorization policies without re-encrypting the data again is a desirable feature of access control service. Homomorphic encryption (Gentry et al. 2009) is a novel approach for privacy preserving in publish/subscribe systems, it supports complex computation conducted on the broker, but is not practical. Compared to the above works, in this paper, the policy anonymity approach based on anonymous sets is applied to realize policy privacy. Blinding and encoding operations on event type and policy are carried out to optimize the performance of matching and storage. Our solution considers that the delegation capabilities and flexible authorization management are both requisite for access control.

### Privacy preserving degree

Privacy issues in publish/subscribe system have been studied for a long time (Shikfa et al. 2009; Pal et al. 2012). However, most prior research on data confidentiality in publish/subscribe systems mainly focuses on the privacy of either subscription or publisher, there has been little work to support a comprehensive privacy protection of the published event (metadata) and the subscribed event types (Onica et al. 2016). Choi et al (2010) adopted the encrypted matching approach and Wun and Jacobsen (2007) adopted the policy management approach to protect the privacy of the published data and the subscribed data. Rao Bacon (2008) and Rao et al. (2013) investigate preserving subscription privacy in publish/subscribe systems, which are limited to supporting fine-grained access control for the published data. Opyrchal et al. (2007) focused on addressing issues of publication privacy in publish/subscribe systems by providing access control on publication. Ion et al. (2010, 2012), Pal et al. (2012) presented privacy-preserving schemes that are used to preserve subscription privacy and confidentiality of the publications. Our work is similar to Ion et al. (2012), Pal et al. (2012), however, these works adopt cryptography encryption to achieve privacy-preserving objects, which limits the efficiency of the privacy-preserving scheme.

The basic security requirements of a wide-area SCADA system over publish/subscribe-based infrastructure, and the solution to meet the requirements are presented in Zhang and Chen (2012). However, that paper did not discuss how to address the policy privacy issue in a two-layer protection way and how to embed authorization policies into events separately. In addition, the policy privacy was not considered, and the key focus was how to adopt an appropriate encryption scheme to provide distributed security framework. This paper is a continuation of the work that was presented in Zhang and Chen (2012), where a complete security framework is given, and the policy attaching issue and policy privacy are thoroughly addressed. Our access control framework is an extension of Zhang and Chen (2013) by adding the description of embedding policy and preserving policy.

## Preliminaries

In this section, a publish/subscribe-based IoT communication infrastructure is modeled. The formal definitions for attribute-based authorization policy are provided. Furthermore, we give background information on the Bloom Filter, which is used to encode attributes and policies.

### Publish/subscribe-based IoT communication infrastructure

**Fig. 1:**
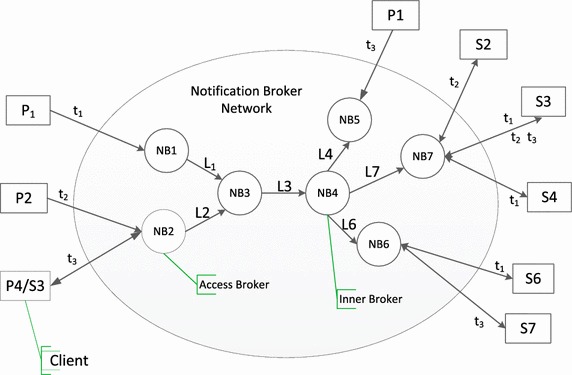
SCADA services communication infrastructure

A publish/subscribe-based IoT communication infrastructure (generally referred to as a Distributed Event-based System) is composed of a set of notification broker (NB) nodes distributed over a network. These NB nodes construct an overlay network, which is a logical network built on top of the physical network as shown in Fig. 1. The nodes of the overlay network are brokers, and their links are paths in the physical network.

Formally the distributed event-driven IoT service communication infrastructure can be modeled as a 5-tuple $CF=\langle B, L, P, S, T\rangle$, where: $B=(NB_1, NB_2, \ldots )$ is the set of notification broker nodes; $L=(L_1, L_2, \ldots )$ is the set of connections between broker nodes; $P=(P_1, P_2, \ldots )$ is the set of publishers that may be some IoT services; $S=(S_1, S_2, \ldots )$ is the set of subscribers that may be other IoT services; and $T=(t_1, t_2, \ldots )$ is the set of event types.

Each publisher (e.g., $P_1$) or each subscriber is connected to only one of the brokers (e.g., *NB*1) in Fig. 1. The notification broker (e.g., *NB*2) that is connected to a subscriber (e.g., $S_1$) (or publisher) is called the access broker from a network view, and is also called the home broker with respect to that subscriber or publisher. The notification brokers that route events between brokers are called event routers or inner brokers (e.g., *NB*4). Each publisher publishes events to its home broker. Each subscriber receives events from its access broker. Clients can be a publisher, or a subscriber, or both.

### Attribute-based authorization policy

In this paper, we adopt the attribute-based access control model (Hu et al. 2015).

#### **Definition 1**

*(Attribute Tuple)* The attribute of a subject *S* is denoted by $s_k=({s_attr}_k, op_k, value_k)$ and the attribute of an object *O* is denoted by $o_e=({o_attr}_n, op_n, value_n)$, where ${s_attr}_k$ and ${o_attr}_n$ are the attribute names, *op* is the attribute operation such as $op \in \{=, <, >, \le , \ge , in\}$, *value* is the attribute value. The action attribute can be one of object’s attributes. The attribute tuple is $\langle s_1, s_2, \ldots , s_K\rangle$ or $\langle o_1, o_2, \ldots , o_N\rangle$, where the relationship among the attributes is conjunction. The subject *S* and object *O* can be represented respectively by the set of attribute tuples $\{\langle s_1, s_2, \ldots , s_K\rangle \}$ and $\{\langle o_1, o_2, \ldots , o_N\rangle \}$.

In our paper, the *op* is simplified as $\{=\}$ by describing digital attributes with careful intervals. Then *S* can be written as $(w_{1,1}\wedge \cdots \wedge w_{1,K_1})\vee \cdots \vee (w_{l,1}\wedge \cdots \wedge w_{l,K_l})$, where $w_{i,j}:=\text{``}s_attr_{i,j}=value_{i,j}\text{''}$, $1\le i\le l,1\le j \le K_l$. *O* can be written as $(w_{1,1}\wedge \cdots \wedge w_{1,N_1})\vee \cdots \vee (w_{n,1}\wedge \cdots \wedge w_{n,K_n})$ , where $w_{i,j}:=\text{``}o_attr_{i,j}=value_{i,j}\text{''}$, $1\le i\le n,1\le j \le N_n$.

#### **Definition 2**

*(Authorization Rule)* An attribute-based authorization rule is *rule* = ($\langle s_1, s_2, \ldots ,s_K\rangle$, $\langle o_1, o_2, \ldots ,o_E\rangle$), the *j*-th subject attribute in *rule* is written as $rule.s_j$, The *j*-th object attribute in *rule* is written as $rule.o_j$.

#### **Definition 3**

*(Authorization Policy)* An authorization policy $AP_i$ is the set of authorization rules, which can be represented as $AP_i=\bigcup \nolimits _{j=1}^L rule_{i,j}$, where $rule_{i,j}$ is the *j*-th element in the rule set $AP_i$.

For example, a company, called *JingFang*, manages the provision of heating for citizens in the winter. The heat consumption data is classified into *A* and *B*. The data of class *A* is the detailed record for heat consumption of each residential home. The data of class *B* is the record for recording the statistical information of heat consumption. *JingFang* publishes these data in the SCADA system. There are two types of clients to access to the data *C*1 and *C*2. The clients of type *C*1 are individuals who can access their home consumption data *A*. The client of type *C*2 is a data mining company serving for *JingFang*, which can access the data of class *B*.

The attributes of these data and clients are as follows:

1. A: $\langle (class,=,individual), (consumer, =, X)\rangle$, where *X* is the detailed identifier of a consumer who consumes the heat and produces the data. For the data of class *A* from different homes, the identifiers are different.
2. B: $\langle (class, =, statistics), (period, =, X1)\rangle$, which indicates that the data are the list of statistical information for head consumption. That is to say, the data have the attributes as: its class is *statistics*, and the statistics period is *X*1.
3. C1: $\langle (type,=,individual), (consumer,=,Y)\rangle$ where *Y* is the detailed identifier of the consumer. That is to say, the subject has the attributes as: its type is *individual*, its consumer identifier is *Y*.
4. C2: $\langle (type, =, company), (service, =,datamining)\rangle$. That is to say, the subject has the attributes as: its type is *company*, its service is *datamining*.

From the above example description, the authorization policy for data *A* can be represented as $AP_A=(\langle (type, =, individual),(consumer, =, Y)\rangle , \langle (class, =, individual), (consumer, =, X)\rangle )$. There is one authorization rule $(\langle (type, =, individual),(consumer, =, X)\rangle$, $\langle (class, =, individual),(consumer, =, X)\rangle )$ in the policy. This means that the client with the attribute $\langle (type, =, individual),(consumer, =, X)\rangle$ can read the data *A* with the attribute $\langle (class, =, individual),(consumer, =, X)\rangle$. In $AP_A$, the subject attribute tuple is $\langle (type, =, individual),(consumer, =, X)\rangle$, the object attribute tuple is $\langle (class, =, individual),(consumer, =, X)\rangle$. Similarly, the authorization policy for *B* is represented as $AP_B=(\langle (type, =, company),(service, =, datamining)\rangle , \langle (class, =, statistics), (period, =, X1)\rangle )$.

Let ${\varGamma }$ be an expression representing the subject attributes of rules in authorization policy required to access some data, which uses logic operators to associate the attributes, also called authorization policy, if there is no confusion. According to the definition of the authorization policy *AP*, ${\varGamma }$ could be represented as ${\varGamma }=(w_{1,1}\wedge \cdots \wedge w_{1,K_1})\vee \cdots \vee (w_{l,1}\wedge \cdots \wedge w_{l,K_l})$, where $w_{i,j}::=\text{``}s_attr_{i,j}=value_{i,j}\text{''}$, $1\le i\le l,1\le j \le K_l$. According to the authorization policy $AP_B$ for data *B*, i.e., $AP_B=\{(\langle (type, =, company),(service, =, datamining)\rangle , \langle (class, =, statistics)\rangle )\}$, the expression for data *B* is ${\varGamma }_B= \text{``}type = company\text{''} \wedge \text{``}service = datamining\text{''}$. If a customer has attributes to match ${\varGamma }$, he/she can access the data *B*. That is to say, the conjunction of the client’s attributes includes the conjunction in ${\varGamma }$ of the data. $\gamma$ often denotes a customer’s set of attribute conjunctions as the authorization policy. For the negative of $w_{i,j}$, we can set another attribute $w'_{i,j}$ to represent it. The subject can be written as $\text{``}type=individual\text{''} \wedge \text{``}consumer = X\text{''}$.

### Bloom filter

A Bloom Filter is a simple, space-efficient randomized data structure for representing a set of strings compactly for efficient membership querying (Bonomi et al. 2006). A Bloom Filter for representing a set $X=\{x_1, x_2,...,x_n\}$ of *n* elements is described by an array of *m* bits, initially all set to 0. A Bloom Filter uses *k* independent hash functions $\{h_1, h_2,...,h_k\}$ with the range $\{1, 2,...,m\}$ . For each member *x* belonging to *X*, the bits $h_i(x)$ are set to 1 for $1\le i \le k$. The bits can be set to 1 multiple times, but only the first change has an effect. After repeating this procedure for all members of the set, the programming of the filter is completed.

The query process is similar to programming. To check if an item *y* is in *X*, we check whether all $h_i(y)$ are set to 1.

## Access control framework for SCADA systems

**Fig. 2:**
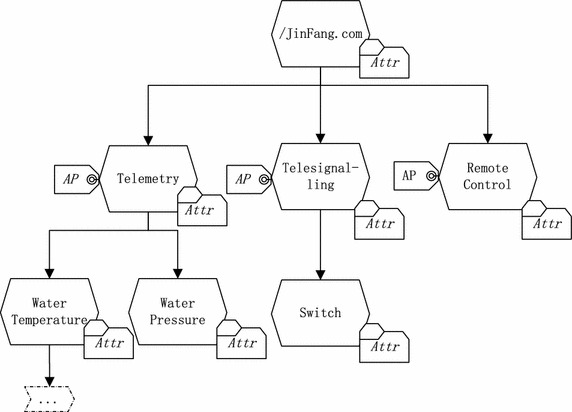
The relation between event names and access control policies

In this section, we present our access control framework for SCADA systems. Our access control framework has two layers, where the bottom layer assumes the matching between the protection requirements of the SCADA events and SCADA applications’ capabilities, and the upper layer assumes the matching between the capabilities of the SCADA events and the SCADA applications’ requirements. The matching function is carried out based on some meta-data such as authorized attributes acting as capabilities and embedded policies acting as requirements. In order to improve the performance of access control schemes, the relation between meta-data and event names is first defined as in Fig. 2. In Fig. 2, *JinFang* is a company that provides heat service for residents in winter. It has a heat provision system that produces and consumes events named Telemetry, Telesignalling, Remote Control, and so on. The Telemetry name has some child names such as Water Temperature, Water Pressure, and so on. Each name in the name tree has its own attributes *Attr*, but an access control policy *AP* is made for one sub-tree such as *Telemetry*. It is worth pointing out that one event name has many name instances, which seems to contradict the assumption of the publish/subscribe paradigm. In our SCADA system, however, if a name has its child names with no different attributes and authorization policies, its child names are only used to tag different data packets and we can regard its child names as its instances. Such a method will obviously reduce the size of the name tree. For example, a sensor continuously measures the temperature of water and publishes the temperature data event every second. Different temperature data have only the difference in timestamp. We can regard all temperature data with different timestamps as different name instances of the same name: Water Temperature. This does not contradict the assumption of SCADA systems (i.e.that each data packet has a unique name) because different data can be further identified by timestamp. That is to say, we can use an instance identifier to further name a data packet, even if the parent name is common. Therefore, we use the concept *type* to handle this scenario. This means that different data packets with the same type may have a common parent name with the same attributes. Multiple types may have a common access control policy. The relation between event names and access control policies is as follows:

1. An event name may have many instances that have the same attributes. That is to say, these instances have the same type. A type is defined by attributes, i.e., a subject attribute expression. It is possible that two event names have the same type. In practice, a type is often unique.
2. Access control policies are often made for sub-trees. Multiple types may have the same access control policy.

**Fig. 3:**
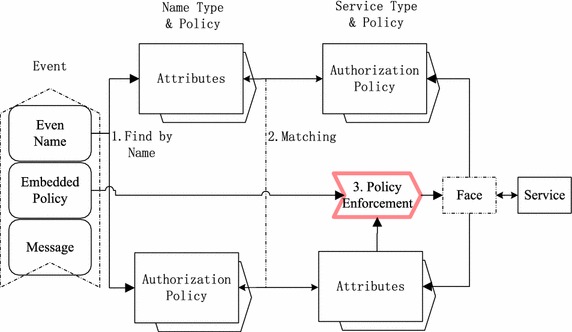
Two-layer framework of access control for SCADA systems

A two-layer framework of access control for SCADA systems is illustrated in Fig. 3. The main component in the framework is an access control engine, a new network entity deployed in home brokers, which lies in the middle column. The engine stores name’s types and policies, as well as services’ types and policies. When a service message arrives at the home node, the engine finds the access control policy and type by event name. It then checks for matching between name type & policy and service type & policy if the consumers subscribe to events with the name in the received event. If the matching results are not empty, the engine will enforce polices in data layer for valid consumers, where the privilege value in the event is the embedded part of the access control policy. The embedded privilege not only binds the access control policy and type to event, but also provides authentication to indicate that only the event publisher can embed such value. The access control in the application layer may be carried out by the service itself. The service can also delegate some responsibilities of access control in the application layer to the engine in SCADA systems.

The engine in the access control framework assumes three functions, which are illustrated in Fig. 3: (a) Finding a name type & policy by name, (b) Matching between requirements and capabilities, and (c) Enforcing policies. In order to realize these functions, two building blocks have to be provided. One is to embed authorization policies and types into service messages, and support dynamically generating and embedding session attributes. The embedding scheme should provide authentication support because the bi-direction matching should be finally verified to have been carried out based on actual attributes. It is desirable that the scheme itself assumes this authentication task for performance optimization. The other is to encode attributes and policies for rapid matching and keeping privacy (Bonomi et al. 2006).

**Fig. 4:**
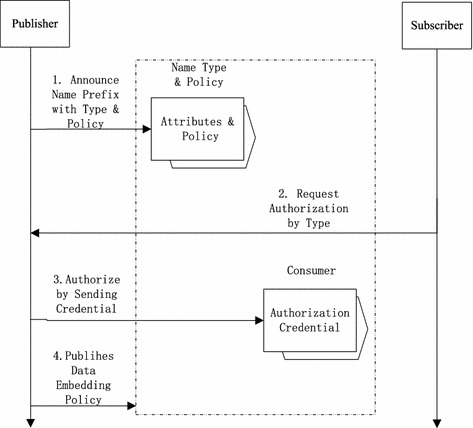
Authorization before disseminating data

Figure 4 illustrates an authorization procedure before the publisher publishes the data (or service messages) in SCADA systems. The detailed steps are as follows:

1. The publisher attaches the name type and access control policy to the data prefix announcement. The access control engine stores the received name type and access control policies in its storage, called *Name Type & Policy.*
2. A subscriber publishes its authorization request for the name by its type.
3. After receiving the authorization request, the publisher translates the name policy into a service policy part, called privilege value, and a network policy part, called authorization credentials. The publisher publishes to the access control engine the network policy part, which means that the SCADA systems cannot disclose some sensitive information, even if the authorization credential is stored in the engine.
4. Embedding the service policy part into data, which will bind the type and policy to the published data.

The authorization procedure is not our focus in this paper, see Zhang and Chen (2013) for further details.

The data consumers trust their home nodes and assume that these home nodes are honest. The data producers assume that the home nodes are honest but curious. That is to say, the home nodes will follow predefined protocols, but will try to find out as much secret information as possible. Home nodes might collude with malicious users. Adversaries control all communication channels, and can eavesdrop, forge, delay and discard messages as well as dynamically corrupt any participants in the system.

## Policy embedding scheme

The policy embedding function and the blind encoding function are the cornerstones of the access control framework. In this section, we give the basic embedding scheme. In the basic scheme, each access control policy is expressed by an access expression ${\varGamma }$ such as

$$\begin{aligned} {\varGamma }=(w_{1,1}\wedge \cdots \wedge w_{1,n})\vee \cdots \vee (w_{l,1}\wedge \cdots \wedge w_{l,l_n}). \end{aligned}$$

where ${\varGamma }$ is a propositional formula, i.e., a disjunctive normal form, $(w_{1,1}\wedge \cdots \wedge w_{1,n})$ is a conjunctive clause, $w_{i,j}$ is a basic proposition such as $attr_{i,j}=value_{i,j}$, i.e., an atomic formula. A type is expressed by a subject attribute expression $\gamma$ such as

$$\begin{aligned} \gamma =(w_{1,1}\wedge \cdots \wedge w_{1,n'})\vee \cdots \vee (w_{l',1}\wedge \cdots \wedge w_{l,l_n'}). \end{aligned}$$

where the subject attributes and object attributes are both represented by *type*, i.e., subject and object being relative.

The goal of embedding type and policy is to compress the variable length of attribute name and value such that it is possible to optimize the performance of matching, communication and storage. The core idea is to adopt the one-way set hash method to encode the attributes in a conjunctive clause, i.e., a set of attributes, of disjunctive normal form into a hash value. In addition, privacy can be considered in embedding. During evaluation of a customer’s subscription for some sensitive event data, directly matching the customer’s clear attributes against authorization policies will result in disclosing some critical information of the customer or the data owner. Thus, we adopt the policy anonymity approach, where the attribute-based access control model is used. Each customer has her/his own attributes, which are disjunctive normal forms of attribute conjunctions such as $(w_{1,1}\wedge \cdots \wedge w_{1,K})\vee \cdots \vee (w_{l,1}\wedge \cdots \wedge w_{l,l_K})$. As the customer, each data event also has its attributes, but we pay attention to the subject attributes in the authorization policy for the data event, which is identified by the data’s attributes. Authorization policies made by the data owner are to say what attributes a customer should have, in order to access the data event. The home broker makes a decision about the customer’s subscription by matching the customer’s attributes against the data’s authorization policy, i.e., checking whether there is an attribute conjunction of the customer including one attribute conjunction of the authorization policy.

In order to clarify the idea of policy anonymity, we give an abstract of an anonymous set according to our requirements. We then use the abstract as a clear and formal basis to design our policy-attaching and policy-privacy scheme. For the abstract of our anonymous set, one-way random functionality and compression functionality, called set hash, play a key role in encoding the attributes in a conjunctive clause, i.e., a set of attributes, of disjunctive normal form into a hash value. The abstract of the anonymous set is defined as follows:

### **Definition 4**

*(Random Oracle*$O_{set}$*for Set)* Given a set of string elements, we obtain a random bit string, which is called Random Oracle for Set, if the conditions below are satisfied.

1. For two different sets, the random bit strings output by the oracle $O_{set}$ are different;
2. For an element in the set, the membership can be checked by the membership checking oracle $O_{\in }$;
3. For a sub-set of the set, the inclusion relation can be checked by the set inclusion oracle $O_{\subseteq }$;
4. For two sets, their union can be computed by the set union oracle $O_{\cup }$;
5. For two sets, their intersection cannot be computed if there exists no inclusion relation;
6. No elements can be computed from the set hash value (i.e.the random bit string) if the set is not publicly known.

According to this definition, a set of sensitive attributes is encoded into one-way string code and member elements are not able to be directly recovered from the code. A Bloom Filter can be used to realize such an oracle $O_{set}$, but it has the deficiencies of privacy as follows:

1. Encoding a clear authorization policy into a Bloom Filter, some sensitive information can be guessed during the evaluation of customers’ requests by testing membership of clear subject attributes. An attribute-blinding method should be adopted to address this issue.
2. After attributes are blinded, a membership-checking function is often used in many scenarios, which is carried out upon an explicitly given blinded attribute. When the blinded attribute is explicitly given during the membership checking, it is also a clue to link different Bloom Filters for different attribute sets, to link authorization transactions, and to guess the corresponding clear attributes, because the membership-checking result indicates whether two attribute sets include the same attribute. Therefore, the blinded attribute should be kept unknown to adversaries.
3. The membership-checking is a basic function of a set. We should propose an alternative way, where, instead of the membership-checking function, the anonymous set-inclusion-checking function is used to answer the membership querying, i.e., using two Bloom Filters to complete anonymous membership querying. To the best of our knowledge, there are no existing algorithms that use set-inclusion-checking function to complete the anonymous checking function of a set member.

Therefore, the policy embedding scheme should be designed based on a Bloom Filter, where the membership-checking function is a key factor of the scheme. When we talk about using the set-inclusion-checking function to assume the membership-checking function, we mean that, for a customer’s attribute conjunction, which attributes of the conjunction are included in a given authorization conjunction can be queried by inclusion queries without explicitly knowing these attributes. That is to say, each attribute in the conjunction is ordered with an index, and we try to find a method to obtain these indices, to which the attributes corresponding satisfy the authorization conjunction. The same index value in different authorization conjunctions may correspond to different attributes. When finding these indices, customers’ attributes and attributes in the policy are not known and disclosed. These indices are often passed into other functions or used as an indictor to say whether they are matched.

The key idea to realize the alternation way for membership-checking function is to sort each attribute conjunction, predefine a series of auxiliary sets for each attribute conjunction of the customer, and then judge which auxiliary sets include one of the attribute conjunctions in the authorization policy. When these auxiliary sets are identified, attributes indices are computed according to the indices of these auxiliary sets. These are described in more detail below:

### **Definition 5**

*(Auxiliary Sets and Attributes Indices)* Assume the number of a customer’s attribute conjunctions is *x* and the number of attributes in a conjunction is *y*, and the size of the Bloom Filter is *m*. We define a series of auxiliary sets for the attributes $$w_1, w_2, \ldots , w_y$$ in a conjunction: $$Set_1=\{w_1, w_2, \ldots , w_{y-1}\}$$, $$Set_2=\{w_1, w_2, \ldots , w_{y-2},w_y\}$$, $$\ldots$$, $$Set_y=\{w_2, \ldots ,w_y\}$$. If there is a set *AWset* that is only included in one $$Set_i$$$$(1\le i \le y)$$ and not included in other sets $$Set_j$$$$(1\le j \le y)$$, then *AWset* includes the attributes as in $$Set_i$$ and these included attribute indices are $$1, \ldots , y-i, y-i+2, \ldots , y$$. If the set *AWset* is only included in two sets $$Set_i$$$$(1\le i \le y)$$ and $$Set_j$$$$(1\le j \le y)$$, and not included in other sets $$Set_k$$$$(1\le k \le y)$$, then *AWset* includes the attributes as in $$Set_i \cap Set_j$$, assume $$j>i$$, when $$j>i+1$$, the attribute indices are $$1, \ldots , y-j, y-j+2, \ldots , y-i, y-i+2, \ldots , y$$; when $$j=i+1$$, the attribute indices are $$1,\ldots , y-j, y-j+2, \ldots , y$$. The remainder can be done in the same manner. If the set *AWset* is only included in the set $$Set_{y+1}$$, and not included in other sets $$Set_i$$$$(1\le i \le y)$$, then *AWset* includes the all attributes as in $$Set_{y+1}$$, and the attribute indices are $$1, \ldots , y$$.

## Policy encoding and matching

Our policy embedding scheme is based on a policy anonymity approach. In our approach, there are three steps to realize policy privacy in the access control service: blinding attributes, encoding blinded attributes into anonymous set, and matching between the customer’s anonymous attribute set and an anonymous authorization policy set.

### Blinding attributes

The first step is to blind attributes, which mainly consists of blinding the data’s attributes, the customer’s attributes, and authorization policies. The procedures are described as follows: 
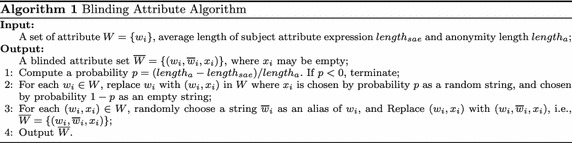

1. Given the set of attributes $W=\{w_1, w_2, \ldots , w_n\}$ from all attribute conjunctions of all customers, a data owner makes authorization policies according to it. The elements of the set *W* are subject attributes. The data attributes can be discussed as the subject attributes and are not discussed further here.
2. For each $$w_i\in W$$$$(1\le i \le n)$$, a string $$\overline{w}_i$$ is randomly chosen as an alias of $$w_i$$, and $$w_i$$ is replaced with $$\overline{w}_i$$. $$\overline{w}_i$$ is kept secret such that all elements in *W* are unknown by the home brokers, clients and adversaries.
3. For each $$\overline{w}_i\in {W}$$$$(1\le i \le n)$$, $$\overline{w}_i$$ is replaced with $$(\overline{w}_i,x_i)$$ in *W*, where $$x_i$$ is chosen by probability *p* as a random string and chosen by probability $$1-p$$ as an empty string. Thus, given an attribute conjunction with length *length* as input, the length of output conjunction varies, where the attribute $$w_i$$ in the attribute conjunction is replaced by $$(\overline{w}_i, x_i)$$ if $$x_i$$ is not empty, or by $$\overline{w}_i$$ if $$x_i$$ is empty.

Through these steps, *W* becomes $\overline{W}$.

We assume that the number of attributes in attribute conjunctions averages out to $$length_{sae}$$, and that the length $$length_{sae}$$ is extended to the anonymity length $$length_a$$ to give each attribute conjunction an anonymity space $$length_a-length_{sae}$$. Algorithm 1 depicts the process of blinding attributes. From Algorithm 1, we know the set of attributes used in the access control service is extended to the $$((length_a-length_{sae})/{length_a+1})$$ times of original one by appending those non-empty attributes $$x_i$$$$(i=1,2,\ldots )$$ to *W*. For each attribute $$w_i$$ in the attribute set *W*, we define its alias as $$\bar{w}_i$$, which is a random string. 
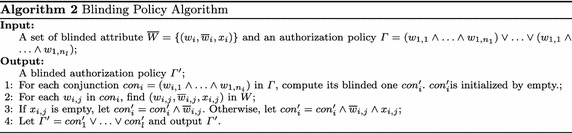


In Algorithm 2, the authorization policy is blinded, where, if an element $w_i$ of the authorization policy has $(w_i, \overline{w}_i, x_i)$ in the blinded attribute set $\overline{W}$ and $x_i$ is not empty, $x_i$ is inserted into the authorization policy. The element $w_i$ is replaced by its alias $\overline{w}_i$ in the expression. The alias and added $x_i$ are not published, and only known by the data owner.

### Policy encoding

When attributes and policies are blinded, the second step is to encode blinded attribute conjunctions from authorization policies and the customer into anonymous sets. The Bloom Filter is used to encode the blinded attributes. The final step is to compute the set membership, the set inclusion and intersection of two anonymous sets of the data and customer. The alternation scheme is designed to use the set-inclusion-checking function to complete the membership querying based on two anonymous sets. If the scheme is available, our anonymous-set-based idea may be used to realize policy privacy.

The *Encoding Procedure* is defined to describe how to obtain predefined auxiliary sets without disclosing clear attributes. The* Matching Procedure* is defined to describe how to identify these auxiliary sets, including the authorization conjunction, and to compute attribute indices without disclosing clear attributes.

#### **Definition 6**

*(Encoding Procedure)* The encoding procedure includes two parts: encoding of the attribute conjunctions of customers, and encoding of the attribute conjunctions of authorization policies.

1. Encoding for customers’ attributes

We expand each attribute conjunction with the number of attributes in the conjunction being *n*, where the random attributes have been inserted into the conjunction to hide the conjunction length (the attributes and attribute conjunctions are also blinded by using Algorithm 1 and Algorithm 2, which are discussed in the next section). This is in Table 1, where the whole Bloom Filter $BF_t$ represents the attribute conjunction, Bloom Filter $BF_1$ represents the first auxiliary attribute set $Set_1$, Bloom Filter $BF_2$ represents the second auxiliary attribute set $Set_2$, and so on.

**Table 1:** Bloom Filters in one attribute conjunction

$BF_t$, $BF_1$, $BF_2$, $\ldots$, $BF_n$	$w_1$	$w_2$	$\ldots$	$w_n$

The attributes in the conjunction are distributed in the Bloom Filters as in Table 2. The row of the table represents the Bloom Filter, and the column represents the attribute. For example, the $i-th$ row represents $BF_i$, and the $j-th$ column represents $w_j$. If $BF_i (1\le i \le n)$ has $\text{``}1\text{''}$ in the $j-th$ column, then $w_j (1\le j \le n)$ is encoded into $BF_i$, i.e., $w_j$ belonging to the $i-th$ auxiliary attribute set $Set_i$. That is to say, if the element (*i*, *j*) in the table is $\text{``}1\text{''}$, then $w_j (1\le j \le n)$ is encoded into $BF_i$. The bottom row, i.e. the $(n+1)-th$ row, represents $BF_t$, where all attributes in the conjunction are encoded into $BF_t$. The right column rounded by dashed line says that each row itself is a bit string, and is denoted by $B_i (1\le i \le n)$. For example, $b_1=\overbrace{11\ldots 10}^{n}$, $b_i=\overbrace{11\ldots 1_{n-i}0_{n-i+1}1_{n-i+2}\ldots 11}^{n}$ and $b=\overbrace{11\ldots 11}^{n}$.

**Table 2:** Attribute distribution among Bloom Filters

	$w_1$	$w_2$	$\ldots$	$w_{n-1}$	$w_n$	*bitstrings*
$BF_1$	1	1	$\ldots$	1	0	$b_1$
$BF_2$	1	1	$\ldots$	0	1	$b_2$
$\ldots$			$\ldots$			$b\ldots$
$BF_{n-1}$	1	0	$\ldots$	1	1	$b_{n-1}$
$BF_n$	0	1	$\ldots$	1	1	$b_n$
$BF_t$	1	1	$\ldots$	1	1	*b*

For the Bloom Filter $$BF_i$$$$(1\le i \le n)$$, it is computed as follows:

1. $BF_i$ is initialized to zero;
2. In the $i-th$ row of Table 2, all attributes with $\text{``}1\text{''}$ in their position form a set $Set_i$;
3. A random string is chosen to put into $Set_i$;
4. $Set_i$ is encoded into a Bloom Filter which is assigned to $BF_i$.

For the Bloom Filter $BF_t$, it is computed as follows:

1. $BF_t$ is initialized to zero;
2. All attributes in the conjunction form a set $Set_t$;
3. A random string is chosen to put into $Set_t$ if no random string is inserted into the conjunction during expanding;
4. $Set_t$ is encoded into a Bloom Filter which is assigned to $BF_t$.

2. Encoding for the attribute conjunction in authorization policies

The Bloom Filter $BF_a$ for the attribute conjunction in an access expression, the mask Bloom Filter $BF_{a-m}$ are computed as follows:

1. $BF_a$ and $BF_{a-m}$ are initialized to zero;
2. All attributes in the conjunction form a set $Set_a$;
3. Some random strings are chosen to be put into $Set_a$, and also form a mask set $Set_{a-m}$;
4. $Set_a$ is encoded into a Bloom Filter, which is assigned to $BF_a$;
5. $Set_{a-m}$ is encoded into a Bloom Filter, which is assigned to $BF_{a-m}$.

From the definition of encoding procedure, we know that each $BF_i (1\le i \le n)$ is encoded from $Set_i=\{w_1, \ldots , w_{(n-i)}, w_{(n-i+2)}, \ldots , w_n\}$ and a random string. The random string is a blinded mask for $BF_i$, which does not affect checking whether an attribute is a member of $Set_i$ and whether an attribute set is included in $Set_i$.

### Policy matching

For the attribute set $Set_a$ of an access conjunction, it is impossible to check whether it is included in the attribute set $Set_t$ of subject conjunction when its Bloom Filter $BF_a$ is blinded . To address this issue, we encode the random strings used for blinding mask into an independent

**Fig. 5:**
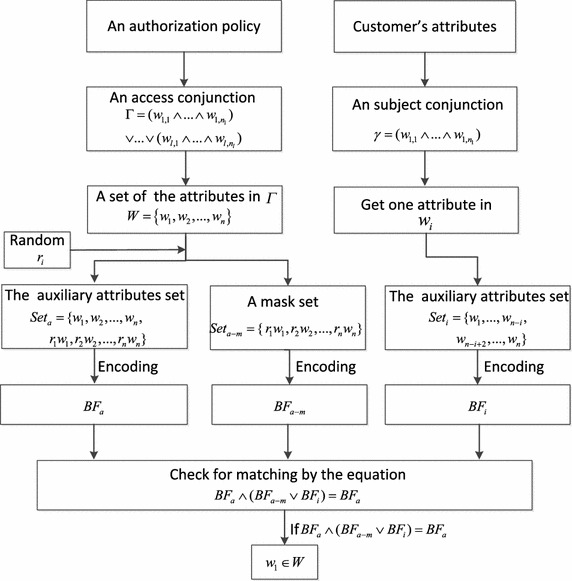
Matching procedure

Bloom Filter $BF_{a-m}$. Because the Bloom Filter is one-way, it is impossible to remove the blinding mask strings, even if $BF_a$ and $BF_{a-m}$ are given. Using bit “OR” operation, $BF_{a-m}$ can be added into $BF_i$, i.e., the blinding mask strings being encoded into $BF_i$. Then, the inclusion relationship is checked by *the equation*$BF_a \wedge (BF_{a-m} \vee BF_i)=BF_a$, i.e., being whether the attribute set $Set_a$ for authorization conjunction is included in the attribute set $Set_t$ for customers’ attribute conjunction, all the procedures is shown in Fig. 5).

#### **Definition 7**

*(Matching Procedure)* Given the Bloom Filter for authorization policies: $$BF_a$$, $$BF_{a-m}$$, the matching scheme is as follows, where each $$\text{``}0,1\text{''}$$ bit string of rows in Table 2 is represented by $$b_i$$$$(1\le i \le n)$$, $$`\wedge '$$ is bit $$\text{``}AND\text{''}$$, and $$`\vee '$$ is bit $$\text{``}OR\text{''}$$.

1. Choose a $\text{``}1\text{''}$ bit string with *n* size: *b*.
2. If $BF_a \wedge (BF_{a-m} \vee BF_i)\ne BF_a$, the Bloom Filter for authorization and customers’ attributes are not matched and the computation halts; otherwise, continue the next step.
3. For $i=1$ to *n*, if $BF_a \wedge (BF_{a-m} \vee BF_i)=BF_a$ , then $b=b\wedge b_i$.
4. If none of $BF_a \wedge (BF_{a-m} \vee BF_i)=BF_a$ happens in (3) and (2), the computation halts; otherwise,
5. The indices of matched attributes are the corresponding positions with $`1'$ in *b*. Those $`1'$ positions are actual column indices in Table 2.

The correctness of the matching procedure is true, because:

1. When $BF_a \wedge (BF_{a-m} \vee BF_i)=BF_a$, it implies that the attribute set denoted by the $i-th$ row of Table 2 includes the attribute set of the authorization conjunction denoted by $BF_a$. The attribute set denoted by the $i-th$ row of Table 2 is written as $b_i$.
2. When $BF_a \wedge (BF_{a-m} \vee BF_j)=BF_a$, it implies that the attribute set denoted by the $j-th$ row of Table 2 includes the attribute set of the authorization conjunction denoted by $BF_a$. The attribute set denoted by the row of Table 2 is written as $b_j$.
3. From (1) and (2), we know that the attribute set of the authorization conjunction denoted by $BF_a$ is included not only in $b_i$ but also $b_j$. That is to say, the set is included in the intersection of $b_i$ and $b_j$. Therefore, we compute $b_i \wedge b_j$ to obtain the subset, including the attribute set of the authorization conjunction.
4. The rows of Table 2 can be used to compute all subsets of attributes in the customers’ attribute conjunction. When $BF_a$ matches against more $BF_xs$, the set denoted by $BF_a$ includes fewer attribute elements.

We give an example to illustrate the correctness of the matching scheme. Assume $Set_a=\{w_1, rw_1, rw_2\}$ and $Set_{a-m}=\{rw_1, rw_2\}$, then $BF_1, BF_2, \ldots , BF_{n-1}$ satisfies $(BF_{a-m} \vee BF_i)=BF_a$. We compute *b* as follows:

From $b=\overbrace{10\ldots 00}^n$, we know that only the position of $w_1$ has $\text{``}1\text{''}$ and it is concluded that the attribute with index 1 (and $w_1$ unknown) is the member of $Set_a$. Assume $Set_a=\{w_1, w_2,rw_1,rw_2\}$ and $Set_{a-m}=\{rw_1,rw_2\}$, then $BF_1, BF_2, \ldots , BF_{n-2}$ satisfies $BF_a \wedge (BF_{a-m}\vee BF_i)=BF_a$. We compute *b* as follows:

$$\begin{aligned} b= & {}\,b\wedge b_1 \wedge b_2 \wedge b_3 \wedge \ldots \wedge b_{n-2} \\= & {} \overbrace{111\ldots 111}^n \wedge \overbrace{111\ldots 110}^n \wedge \overbrace{111\ldots 101}^n \wedge \overbrace{111\ldots 011}^n \wedge \ldots \wedge \overbrace{110\ldots 111}^n = \overbrace{110\ldots 000}^n \end{aligned}$$

From $\overbrace{110\ldots 000}^n$, we know that only the position of $w_1$ and $w_2$ ($w_1$ and $w_2$ not exposed) has $\text{``}1\text{''}$ and that $w_1$ and $w_2$ are members of $Set_a$.

The matching function is efficient, because only a simple bit operation is carried out. If the matching function returns *False*, the customer’s subscription is rejected. If the matching function returns *True*, the re-encryption component may be invoked with the matched results from the matching function as an input to indicate what re-encryption keys should be used by the indices.

## Policy privacy

A subscriber can successfully access the requested event only its attributes match the publisher’s authorization policy, the subscriber can accept the subscribed event from the published event type only the event attributes match the subscriber’s authorization policy. Thus our access control solution is correct. In this section, we try to clarify that, no matter what form the attacks take from adversaries, our scheme keeps privacy.

### Policy privacy analysis

The Two-layer access control framework keeps privacy, which is performed through defining the concept of policy privacy and privacy proof. Home brokers are assumed to be semi-honest. This means that they follow predefined protocols while they try to find out as much secret information as possible. Home brokers might not collude with malicious users, but arbitrarily send any information to users. Given such a privacy assumption, we first introduce the definition ${\varPi }_{PE}$ of policy evaluation scheme, and then define the policy-privacy model for ${\varPi }_{PE}$.

#### **Definition 8**

*(Policy Evaluation Scheme*${\varPi }_{PE}$*.)*${\varPi }_{PE}$ consists of four algorithms as follows:

1. *Init* On input the attribute set *W* of a customer and an authorization policy ${\varGamma }$, the blinding attribute algorithm and the blinding policy algorithm generates the blinded attribute set $\overline{W}$ and the blinded policy ${\varGamma }'$ respectively.
2. $EncodeForPolicy ({\varGamma }_i[y])$ On input the $y-th$ attribute conjunction in an authorization policy ${\varGamma }_i$ of the data owner *i*, it outputs some randomized code $BF^\mathcal {P}_i[y]$ and $BF^\mathcal {A}_{i-m}[y]$ by invoking *Encoding Procedure*.
3. $EncodeForAttributes(\gamma _j[x])$ On input the $x-th$ attribute conjunction in the attribute expression $\gamma _j$ of the customer *j*, it outputs some randomized code $BF^\mathcal {A}_j[x]$ by invoking * Encoding Procedure*.
4. $MatchinginPEP(BF^\mathcal {A}_j[x],~BF^\mathcal {P}_i[y])$ On input attribute codes $BF^\mathcal {A}_j[x]$, $BF^\mathcal {A}_{i-m}[y]$ and $BF^\mathcal {P}_i[y]$, it outputs whether two codes are matched by invoking *Matching Procedure*. If the algorithm outputs a negative result, the access request of the customer is rejected.

A policy evaluation scheme ${\varPi }_{PE}$ in the access control system is Chosen-Plaintext Attack (CPA) policy-privacy if adversaries cannot win with a non-negligible advantage, the game is defined as follows:

#### **Definition 9**

*(Non-intersection CPA for*${\varPi }_{PE}.$) For the policy evaluation scheme ${\varPi }_{PE}$ and a probabilistic polynomial time adversary *Adv* running in two phases, it is policy-privacy if *Adv*’s advantage is negligible in the following game:

*Setup*: The challenger invokes the *Init* algorithm of ${\varPi }_{PE}$.

*Training Phase 1*: The adversary is allowed to issue queries for the following oracles:

1. Queries $O_{Encode}$ oracle for *EncodeforAttributes* and *EncodeforPolicy* of ${\varPi }_{PE}$. That is to say, choosing one subject attribute conjunction $A_1$ and one attribute conjunction in an authorization policy ${\varGamma }_1$, outputting encoded attributes $BF_1^{\mathcal {A}}$ and encoded policy $BF_1^{\mathcal {P}}$.
2. Queries $O_{Match}{(BF_1^{\mathcal {A}}, BF_1^{\mathcal {P}})}$ oracle for *MatchinginPEP* of ${\varPi }_{PE}$.

*Challenge Phase* The adversary *Adv* submits two random attribute conjunctions in two authorization policies ${\varGamma }_0$, ${\varGamma }_1$ and an subject attribute conjunction *A*. The challenger flips a random coin $\delta \in \{0,1\}$, and outputs a randomized code $BF_\delta ^{\mathcal {P}}$ to the adversary. No attribute conjunctions ${\varGamma }_0$, ${\varGamma }_1$ have appeared in the previous queries.

*Training Phase 2* Training phase 1 is repeated exactly, except that the adversary may not query *MatchinginPEP*, for $BF_\delta$, not query oracles with any element in ${\varGamma }_0$, ${\varGamma }_1$.

*Guess* Finally, the adversary outputs their guess $\delta '\in \{0,1\}$, and wins the game if $\delta '=\delta$.

The probability is over the random bits used by the challenger and the adversary, where *Adv* makes at most polynomial queries to the oracles.

This definition implies that:

1. For two attribute conjunctions, the adversary cannot distinguish their encodings, i.e., they are unable to link a Bloom Filter to a specific attribute conjunction.
2. The *Non-intersection* requires that any element in the challenge sets ${\varGamma }_0$ and ${\varGamma }_1$ should not have appeared or will not appear in other queries. This indicates that our scheme ${\varPi }_{PE}$ has weaker security than that under CPA.

#### **Definition 10**

*(PRF CPA ASSUMPTIOM)* Given a pseudo-random function *PRF*(*seed*, *key*, *input*) with *seed*, *key* being secretly set, and two attribute conjunctions, *PRF*(*seed*, *key*, *input*) chooses one attribute conjunction and returns one random number, and then it is hard to determine which attribute conjunction is chosen according to the returned random number without knowing *seed*, *key*.

#### **Definition 11**

($PRF\_BF$*Scheme*) A Bloom Filter *BF* is initialized to zero, and a key and *n* seeds are secretly generated. Given an attribute set *eSET*, it invokes *PRF*(*seed*, *key*, *input*) for each attribute $e\vdash eSET$ as input with *n* different seeds to obtain *n* random numbers that are in (0, *m*], i.e., being greater than 0 and less than $m+1$. The position in *BF* is set 1 if one value of *n* random numbers points to it. When all attributes in *eSET* are iterated, *BF* is output.

#### **Lemma 1**

*The*$PRF\_BF$*scheme is CPA-secure if each element in the challenge set is not queried on.*

The conclusion is straightforward. In the security proof, multiple random numbers for one element of the challenge set can be seen as multiple oracle queries for the element during a CPA-Security game, where the oracle answers each query with attaching fixed different numbers to the queried element as different inputs. The random numbers for multiple elements in the challenge set can be seen as multiple oracle queries for different elements. The premise that each element in the challenge set is not queried indicates that, during the challenge of $PRF\_BF$, no queried elements are challenged. It is natural to require that any element in the challenged set will not be queried after challenging.

#### **Theorem 1**

*PES*${\varPi }_{PE}$*is non-intersection CPA policy-privacy*.

#### *Proof*

Suppose algorithm *B* is given a private key, it also generates a series of seeds for random generation. *B* initializes the $PRF\_BF$ scheme with the key and seeds.

*Init* Given a set of attributes $W=\{w_1, w_2, \ldots , w_n\}$, *B* generates a random string $\overline{w}_i$ for each attribute $w_i\in W$, and randomly generates $\overline{w}_i'$ according to the probability *p*. Replacing $w_i$ with $(w_i, \overline{w}_i, \overline{w}_i')$ , we will obtain a new blinded set of attributes $\overline{W}=\{(w_1, \overline{w}_1, \overline{w}_1'), (w_2, \overline{w}_2, \overline{w}_2'), \ldots , (w_n, \overline{w}_n, \overline{w}_n')\}$.

*SetupB* maintains a set hash list $H^{list}$, which is initially empty, and responds to the random oracle queries for *Adv* as described below.

1. Random oracle for a set $H(w_1,\ldots , w_n)$: If this query already appears on the $H^{list}$, then returns the predefined value. Otherwise, the query invokes the $PRF\_BF$ scheme with the set of $\{w_1,\ldots , w_n\}$ to get a Bloom Filter $bf$. $H(w_1,\ldots , w_n)=bf$ is defined. Finally, it adds the tuple $(\{w_1,\ldots , w_n\}, bf )$ to the list $H^{list}$ and respond with $H(w_1,\ldots , w_n)$.
2. $O_\in (BF, w)$: If *BF* can be found in $H^{list}$ with $BF=bf$ in $(\{w_1,\ldots , w_n\}, bf )$ and $w \in \{w_1,\ldots , w_n\}$, then returns true, otherwise returns false.
3. $O_\subset {(BF_1^{\mathcal {A}}, BF_1^{\mathcal {P}})}$: If $BF_1^{\mathcal {A}}$ and $BF_1^{\mathcal {P}}$ cannot be found in $H^{list}$ with $BF_1^{\mathcal {A}}=bf _1$ in $(\{w^1_1,\ldots , w^1_n\}, bf _1)$ and $BF_1^{\mathcal {P}}=bf _2$ in $(\{w^2_1,\ldots , w^2_n\}, bf _2)$, then returns false. Otherwise, if $\{w^1_1,\ldots , w^1_n\}\subseteq \{w^2_1,\ldots , w^2_n\}$, then returns true, otherwise returns false.

*Phase 1* In this stage, the adversary *Adv* issues a series of queries, which are subject to the restrictions of the Non-Intersection-CPA game. *B* maintains a list $K^{list}$ that is initially empty.

1. Encoding Query $$O_{Encode}(w_1, \ldots , w_l)$$$$(l\le n)$$: Algorithm *B* finds the corresponding $$\overline{w}_i,\overline{w}_i'$$ for each $$w_i\in \{w_1, \ldots , w_l\}$$ in *W*, and obtains a new set $$sT=\{\overline{w}_i,\overline{w}_i',\ldots \}$$. If the cardinality of the set *sT* is less than the parameter *k*, some random bit strings are generated and are added into *sT* such that the cardinality of *sT* is equal to *k*. Finally, adds the tuple $$(\{w_1, \ldots , w_n\}, sT, H(sT))$$ to the list $$K^{list}$$ and responds with *H*(*sT*).
2. Matching Query $O_{Match}{(BF_1^{\mathcal {A}}, BF_1^{\mathcal {P}})}$: If $BF_1^{\mathcal {A}}$ and $BF_1^{\mathcal {P}}$ cannot be found in $K^{list}$ with $BF_1^{\mathcal {A}}=H(sT_1)$ in $(\{w^1_1, \ldots , w^1_{l1}\}, sT_1, H(sT_1))$ and $BF_1^{\mathcal {P}}=H(sT_2)$ in $(\{w^2_1, \ldots , w^2_{l2}\}, sT_2, H(sT_2))$ , then returns false. Otherwise, if $\{w^1_1, \ldots , w^1_{l1}\}\subseteq \{w^2_1, \ldots , w^2_{l2}\}$ , then returns true, otherwise returns false.

*Challenge* When *Adv* decides that Phase 1 is over, *Adv* chooses two random attribute conjunctions in authorization policies ${\varGamma }_0, {\varGamma }_1$ and an attribute conjunction *A*. *B* responds as follows:

1. Finds the corresponding $\overline{w}_i, \overline{w}_i'$ for each $w_i$ of ${\varGamma }_0$ and ${\varGamma }_1$ in the blinded attribute set $\overline{W}$, and keeps $w_i$ unchanged if no $\overline{w}_i,\overline{w}_i'$ in $\overline{W}$, then obtains two new sets $sT_0=\{\overline{w}_i,\overline{w}_i',\ldots \}$ and $sT_1=\{\overline{w}_i,\overline{w}_i',\ldots \}$. We simply assume that $sT_0$ and $sT_1$ have the same cardinality (otherwise, padding with random strings ). At the same time, finds the corresponding $\overline{w}_j$ for each $w_j$ of *A* in the blinded attribute set *W*, then then gets two new sets $sT'=\{\overline{w}_i,\overline{w}_i',\ldots \}$.
2. *B* chooses $\delta \in \{0,1\}$ and submits $sT_0$, $sT_1$ and $sT'$ as a challenge to $PRF\_BF$, i.e., sends $BF_1^{\mathcal {A}}$ and $BF_\delta ^{\mathcal {P}}$ as a Matching Query. Assuming that $O_{Match}{(BF_1^{\mathcal {A}}, BF_\delta ^{\mathcal {P}})}$ are the returned results, *B* sends it to *Adv*.

*Phase 2*: The phase 1 is repeated exactly, except that the adversary may not query oracles with any element in ${\varGamma }_0, {\varGamma }_1$ and *MatchinginPEP* for $BF_\delta$.

*Guess*: Eventually, the adversary *Adv* returns a guess $\delta '\in \{0,1\}$ to *B*. *B* also outputs $\delta '$ as the guess of $\delta$ for $PRF\_BF$ game.

If *Adv* has a non-negligible probability $\varepsilon$ in making a successful guess, i.e., guess $\delta '=\delta$. It indicates *Adv* has another non-negligible probability in distinguishing $BF_0^P$, $BF_1^P$, which contradicts the fact that $PRF\_BF$ scheme is CPA security. Thus, we reach a contradiction. $\square$

### Privacy management

Based on the policy embedding scheme ${\varPi }_{PE}$, the authorization management becomes efficient and simple. The policy-privacy authorization management includes Customer Grant, New Subscription Authorization, Authorization Update, and Customer Revocation.

*Customer Subscribing Grant* When a new customer *B* subscribing him or herself to the SCADA system *A*, the system uses the traditional authorization administration tool to decide whether customer *B* is granted. If *B* can be granted, *A* computes as follows:

1. It converts *B*’s subject attribute expression $\gamma$ into a blinded one $\gamma '$ according to ${\varPi }_{PE}$.
2. It encodes $\gamma '$ by the encoding procedure in definition $(\{BF_t, BF_1, BF_2,\ldots , BF_n\})$.
3. It sends corresponding attribute Bloom Filters $(\{BF_t, BF_1, BF_2,\ldots , BF_n\})$ to *B*’s home brokers the access control service.

*New Event Grant* When a new event is published in the SCADA system, it extracts the authorization expression ${\varGamma }$ from the authorization policies. It then computes as follows:

1. It converts the authorization expression ${\varGamma }$ into a blinded one ${\varGamma }'$ according to ${\varPi }_{PE}$.
2. Each conjunction $coin'$ in ${\varGamma }'$ is encoded into Bloom Filters $BF_a$ and $BF_{a-m}$.
3. It sends the corresponding Bloom Filters $\{(BF_a, BF_{a-m})\}$ to the home brokers.
4. Only a hash indicator is attached to the published event. If the encoding policies have been sent for this event type, no policy conversion and transmission take place.

*Authorization Update* When a SCADA application modifies the authorization policy for the event type that it will publish, the access control system computes new Bloom Filters $BF_a'$, $BF_{a-m}'$ according to the new authorization policy. It then sends $BF_a'$, $BF_{a-m}'$ to the home brokers to replace $BF_a$, $BF_{a-m}$ .

*Customer Revocation* When the access control system revokes some privilege of the customer *B*, it computes new Bloom Filters $\{(BF_t', BF_1', BF_2',\ldots , BF_n')\}$. It sends $\{(BF_t', BF_1', BF_2',\ldots , BF_n')\}$ to the home brokers to replace $\{(BF_t, BF_1, BF_2,\ldots , BF_n)\}$.

## Storage cost and performance evaluation

Section “Policy privacy” described the security analysis, which proved that the proposed policy evaluation scheme ${\varPi }_{PE}$ is CPA policy-privacy. In this section, we analyze the false positive probability of Bloom Filter and ${\varPi }_{PE}$’s cost, and then evaluate the communication performance, scalability and policy matching efficiency in the publish/subscribe system with two-layer access control framework in different test cases.

### Storage cost

We analyze the false positive probability of the Bloom Filter and ${\varPi }_{PE}$’s cost because the false positive probability is neglected during proof. Let the length size of a Bloom Filter *BF* be *m*, the cardinality of an element set be *n*, and the number of the hash functions be *k*, then the probability *p* of a random bit being $\text{``}1\text{''}$ in *BF* is $p=(1-1/m)^{n\times k}\approx e^{-nk/m}$. The false positive probability $p_f$ of *BF* is $p_f=(1-p)^k\approx (1-e^{-nk/m})^k$.

Let the number of attributes in a conjunction of authorization policy be *x*, then the false positive probability of *x* attributes in $BF_t$ and $BF_a$ is $p_{BF}=p_f^x+p_f^{x-1}(1-p_f)+\ldots + p_f^1(1-p_f)^{x-1}$. For $BF_1, BF_2, \ldots , BF_n$ , the false positive probability to check whether $BF_a$ is included is $p_{vector}=p^y_{BF}+p^{y-1}_{BF}(1-p_{BF})+\ldots +p^1_{BF} (1-p_{BF})^{y-1}$, where if $x>=n$, then $y=1$, otherwise $y=n+1$.

For example, the average number of attributes in one conjunction is 30, the average number of conjunctions for a customer is 50, and the false positive probability is $< 10^{-10}$ with $0.6185^{\rm{m/n}}$, then the bit size for each conjunction is 1500 with $0.6185^{1500/30}=3.69\times 10^{-11}$, the byte size for a matrix is $1500/8*32=6000\approx 6\,{\rm{KB}}$, and the byte size for a customer is $500*6\,{\rm{KB}}=300\,{\rm{KB}}$. That is to say, the home broker should provide 300 KB storage to store his/her attribute information for a customer. As for the publisher’s attributes, the storage needed for each rule for a data event is $0.187\,{\rm{KB}}=187{\rm{bytes}}$, and that for whole policy for the data event is 9 KB. If the number of attributes in a conjunction is less, then the storage cost will be significantly reduced.

### Performance evaluation

Access control policy enforcement may introduce the overheads for the overall communication performance in publish/subscribe system. In this section, we focus on evaluating (1) the overhead of data event communication performance from publishers to subscribers; and (2) policy matching efficiency via the broker; (3) the scalability of the SCADA system with our access control framework, which is implemented based on a message-oriented Java Message Service (JMS) broker; and (4) the performance impact on overall performance.

*Evaluation Metrics* In order to evaluate the communication performance, scalability and policy matching efficiency in SCADA system with our access control framework, *latency* and *throughput* are used as the performance metrics. Here, two kinds of latencies are considered: *pub-to-sub latency* and *broker latency*. To avoid ambiguity, we present the definitions of these metrics as follows:

1. *Pub-to-sub latency* refers to the total time spent by a data event from its publisher to its subscriber including the time taken for broker matching.
2. *Broker latency* is defined as the time spent by a broker in receiving the published event, performing matching operations against all the requested subscribers and outgoing the data event to the matching subscribers.
3. *Throughput* is defined as the average number of the published data events per second.

**Fig. 6:**
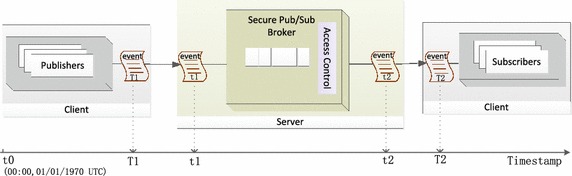
Testing design

*Test Design* We extended Apache ActiveMQ, i.e., one JMS broker, by building in a two-layer access control framework used to preserve policy privacy for the publish/subscribe system. The implementation framework is shown in Fig. 6. The broker connected to the publishers provides the subscribe filters by building the policy-based access control (AC) scheme of the published event. The broker connected to the subscribers provides the publish filters, which are the authorization policies of subscribe services. Such a broker is called *secure pub/sub broker*, which conducts matching operations between the encoded attributes and the encoded authorization policy for each data event. In our test, we used a data event without the authorization policy as the baseline. This means that we do not apply access control (AC) framework on the broker. Such a publish/subscibe system without secure broker is called the publish/subscibe system with plain, in which a publisher publishes the events to his/her broker, the subscribers subscribe events (by event type) through her broker, and the broker sends the data event whose event type matches the subscribed event to the subscriber.

Based on the latency measure method in Chen and Greenfield (2004), the three partial time is measured, which consists of the time from publishing data event to broker, the broker matching time and the time of receiving event from the subscriber’s broker. The detailed procedures of measuring latency shown in Fig. 5 are as follows: the publisher obtains a timestamp $T_1$ and attaches it to the published data event as soon as he/she sends the event to the broker. A broker connected to the publisher receives the event; the broker obtains the $t_1=T_2$. After the broker carries out matching operations, its outgoing data event is attached to the timestamp $t_2$. When the subscriber receives the data event from his/her broker, they obtain the timestamp $T_2$. Pub-to-sub latency can be calculated as $pub{-}to{-}sub latency = T_2-T_1$, broker latency (i.e. matching latency) can be calculated as $broker latency = t_2-t_1$. For simplicity, we assume that the time spent in sending an event from a publisher to the broker is the same as that sending the event from the broker to the subscriber. Therefore, we obtain

$$\begin{aligned} pub{-}to{-}broker \, latency&= broker{-}to{-}sub \, latency\\&= \frac{pub{-}to{-}sub \, latency{-}broker \, latency}{2} \\&= \frac{(T_2-T_1)-(t_2-t_1)}{2}. \\ pub{-}to{-}broker \, latency&= broker{-}to{-}sub \, latency \\&= \frac{pub{-}to{-}sub latency - broker latency}{2} \\&= \frac{(T_2-T_1)-(t_2-t_1)}{2}. \end{aligned}$$

*Test Cases* For the purpose of evaluating the performance property of the publish/subscibe system with two-layer access control framework (PS-ACF), we measure these latency metrics in PS-ACF and baseline (i.e., publish/subscibe system without access control). The test cases are specified as follows:

1. Evaluating latency with access control policy and latency with plain;
2. Evaluating latency metric while the data event size increases;
3. Evaluating latency metric while the number of rules in a policy (i.e., the number of attribute conjunctions in one authorization policy) increases.

The detailed test parameters (e.g., data event size, number of rules) are shown in Table 3. Due to high latency problems for small message sizes (i.e., payloads) of ActiveMQ, in test cases (2), we measure latencies for encoded data event with the large sizes of 1 KB, 4 KB, 16 KB, 64 KB, 256 KB and 1 MB. In order to assure the accuracy of the measured results, each case test is run 1000 times.

**Table 3:** Test cases

Test cases	Description	Event size	Number of rules
1.	For 1 KB data event size, evaluate the latencies with or without AC (number of rules is 1)	1 KB	1
2.	Evaluate the latencies with or without AC (number of rules is 1) for the period 1 KB–1 MB of data event size	1 KB 16 KB 64 KB 256 KB 1 MB	1
3.	For 1 KB data event size, evaluate the latencies with AC for 1–64 rules in a policy)	1 KB	1 4 16 64

**Fig. 7:**
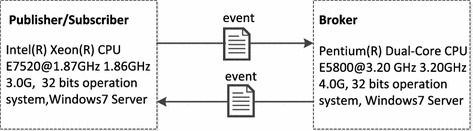
Testing environment

*Test Setup* All experimental tests are carried out using a distributed setup, the configuration of which applied to our experiment is shown in Fig. 7. Since it is difficult to evaluate precisely in a pub/sub system without the same and synchronous clocks, both subscriber clients and publisher client applications (*Publisher/Subscriber*) run on the same computer equipped with $3.0 G$ of RAM and Intel $1.87 GHz$ CPUs running on the $Windows\_7$ 32*bits* operating system; the broker (*Broker*) ran on another server equipped with $4.0 G$ of RAM and Intel $3.2 GHz$ CPUs, $Windows\_7$ 32bits operating system. The publisher/suscriber and broker machines are connected via a standard $100_{\rm{Mbps}}$ LAN.

Based on performing each test case, we obtain the corresponding test results as follows:

**Fig. 8:**
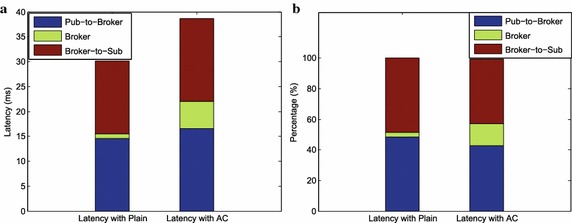
Latencies with plain (1 KB) & access control AC (1 policy)

*Test Results (1)* Test case (1) was carried out by sending 1 KB size data events from one publisher and by adding one access control policy in the broker. In this case, the test results are shown in Fig. 8a, b. Figure 8a shows the latencies spent at each step, comparing to the baseline; the Pub-to-Sub latency is increased by 8ms, the broker latency with AC is increased from $2\, \%$ to $5\,\%$. The broker latency with plain (baseline) is low, and data event is encoded as the random number, which makes broker matching time small; the percentage of broker latency is also low. The Pub-to-Broker time is the same as the Broker-to-Sub time; and the latencies increase by $6\,\%$ when we add one access control policy to the broker.

**Fig. 9:**
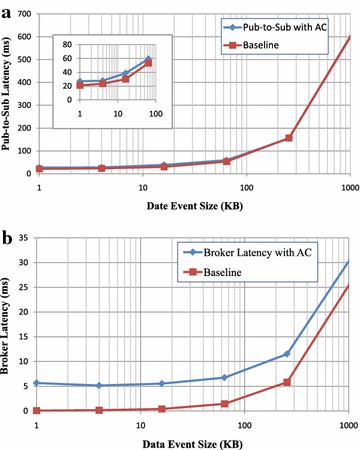
Performance comparison: Pub-to-Sub latency (a) and Broker latency (b) for the PS-ACF and baseline over varying data event size

*Test Results (2)* Test case (2) was carried out by increasing data event size and by adding one access control policy to the broker; results are shown in Fig. 9a, b, and the horizontal axis is logarithmic (base 10). We make a performance comparison between the pub-to-sub latency with plain and with access control, as well as the broker latency with plain and with access control. For small data event sizes, the pub-to-sub latency and broker latency are low, such as for the 1 KB data event size, and the whole latency event messaging latency takes less than 20 ms (Fig. 9a); the policy matching latency taken on the broker takes 5 ms (Fig. 9b). As the data event size becomes larger, the latency is continuous curve. PS-ACF shows the same behaviour as the baseline. As with the pub-to-sub latency and the broker latency, the data event size is one of factors in the overhead.

**Fig. 10:**
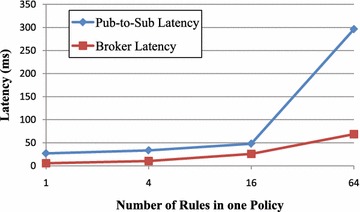
Latencies with different numbers of policy rules

*Test Results (3)* The latencies with the number of policy rules on the horizontal axis are shown in Fig. 10, for a small number of rules (i.e. fewer than 16). Both pub-to-sub latency and broker latency increase slowly with increasing the number of policy rules. For the larger number of rules, the data event messaging time dominates the broker matching time. For 16 rules in a policy, the whole latency event messaging latency takes less than 25 ms and the policy matching latency taken on the broker takes 40 ms. However, the broker latency increases slowly with increasing number of rules, which indicates that our two layers access control framework in the publish/subscribe system is highly scalable and supports matching operations of more policy rules.

*Analysis Results* The collected latency metrics consist of maximum latency, minimum latency, average latency and latency distribution. We present the event latency statistical results based on our measurement metrics in Table 4. The results show that the test running at lower data event sizes, or with fewer policy rules may have lower pub-to-sub latencies and lower broker latencies; furthermore, the spread of latencies is compactly distributed.

**Table 4:** Event latencies in milliseconds (ms)

Event size (KB)	Event latency (ms)	Pub-to-sub (with plain)	Pub-to-sub (with AC)	Broker (with plain)	Broker (with AC)
1 KB	Min	15.699	21.094	0.044	2.328
	Ave	21.100	27.014	0.074	5.647
	Max	131.058	397.548	0.262	81.345
16 KB	Min	23.517	26.017	0.182	2.552
	Ave	30.085	38.658	0.411	5.526
	Max	250.498	281.554	8.338	43.812
64 KB	Min	39.572	42.045	0.620	3.183
	Ave	53.302	59.383	1.438	6.737
	Max	250.426	281.575	4.732	13.999

**Fig. 11:**
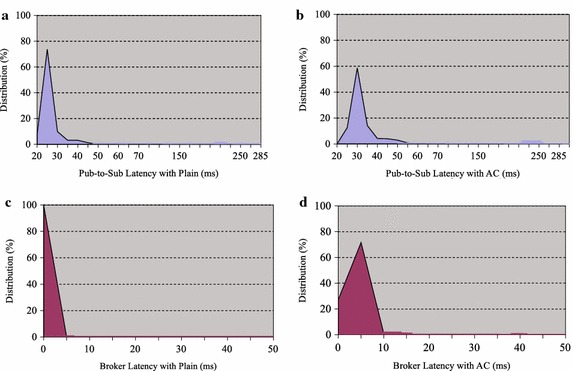
Latency distribution (data event size 1 KB)

The latency distribution test results for a data event size (1 KB) are presented in Fig. 11. As shown in Fig. 11a, b, for 1 KB data event, about $70\,\%$ pub-to-sub latencies with plain are compactly distributed in the range of $25 \sim 30~{\rm{ms}}$. About $55\,\%$ pub-to-sub latencies with one policy are compactly distributed in the range of $30 \sim 35~{\rm{ms}}$. These latency distributions show that the publish/subscribe system with the access control framework presented in our paper has higher throughput and shorter latencies. As shown in Fig. 11c, d, for 1 KB data event, About $95\,\%$ broker latencies with plain are compactly distributed in the range of $0\sim 5~{\rm{ms}}$. About $80\,\%$ broker latencies with one policy are compactly distributed in the range of $5 \sim 10~{\rm{ms}}$. During the tests of all the cases, the CPU utilization was between $15\sim 50\,\%$.

**Fig. 12:**
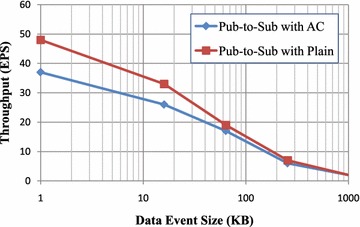
Throughput for different data event size in KB

According to the “Little Law”, we can derive the throughput in Events Per Second (EPS) as $\text{``}Throughput=\frac{1}{Latency}\text{''}$. The pub-to-sub throughput results are presented based on the average pub-to-sub latencies with or without access control. Figure 12 shows the average sustainable throughput in processing events per second using different event sizes; the horizontal axis is given in base-10 logarithms. As with pub-to-sub latencies, the data event size is the main factor in the baseline. With data event sizes increasing, pub-to-sub throughput decreases, that is to say, fewer data events per second can be sent from the publisher to the subscriber.

From the above security analysis and latency evaluation results, the overhead in terms of the number of policies for preserving the publish/subscribe system is easy to observe, but the overhead is reasonable and acceptable. The overall latency comparison shows that our access control framework has higher policy matching efficiency and higher scalability.

## Conclusions and future research

In SCADA systems, named, signed and potentially encrypted content forms a solid foundation for routing and application security. The access control mechanism for SCADA systems should include independent data and application layers; and the two layers should be opaque to network entities as well as be suitable for SCADA communication features, such as event named, caching, and so on. We then propose a two-layer framework of access control for SCADA systems, where, integrating network capabilities, the data layer assumes the protection of the SCADA events, and the application layer assumes the protection of services. The anonymous-set-based principle is adopted to design our policy embedding scheme, which is presented as the foundation of access control service with policy privacy. In our scheme, the alternation method plays a key role, which uses the anonymous set-inclusion-checking function to assume the basic function of the anonymous set, i.e., the anonymous set-membership-checking function. We also extended the open source Apache ActiveMQ broker by adding authorization policies to help realize policy privacy. The evaluation results of latency indicate that our approach is highly scalable and flexible. The security analysis and performance evaluation results of latency show that the SCADA application with our two layers access control scheme flexibly authorizes as in traditional access control systems, and that home brokers can securely and efficiently execute the delegated policy enforcing function without re-encrypting data after the authorization policies are updated, where policies are encoded with blinded mask and are anonymously matched to realize policy privacy. Future research is to make our policy embedding scheme be able to resist more powerful privacy attacks from adversaries.

## Footnotes

1 www.omg.org.

2 http://docs.sun.com/app/docs/doc/816-5904-10.

3 http://www.gridstat.net/trac/.
